# Organ-specific learning curves of sonographers performing first-trimester anatomical screening and impact of score-based evaluation on ultrasound image quality

**DOI:** 10.1371/journal.pone.0279770

**Published:** 2023-02-02

**Authors:** Francesca Bardi, Merel Bakker, Ayten Elvan-Taşpınar, Monique J. A. Kenkhuis, Jeske Fridrichs, Marian K. Bakker, Erwin Birnie, Caterina M. Bilardo

**Affiliations:** 1 Department of Obstetrics and Gynecology, University Medical Center Groningen, University of Groningen, Groningen, The Netherlands; 2 Department of Genetics, University Medical Center Groningen, University of Groningen, Groningen, The Netherlands; 3 Department of Obstetrics and Gynecology, Amsterdam University Medical Centers, Amsterdam, The Netherlands; University of Chicago Medical Center: The University of Chicago Medicine, UNITED STATES

## Abstract

**Introduction:**

First-trimester anatomical screening (FTAS) by ultrasound has been introduced in many countries as screening for aneuploidies, but also as early screening for fetal structural abnormalities. While a lot of emphasis has been put on the detection rates of FTAS, little is known about the performance of quality control programs and the sonographers’ learning curve for FTAS. The aims of the study were to evaluate the performance of a score-based quality control system for the FTAS and to assess the learning curves of sonographers by evaluating the images of the anatomical planes that were part of the FTAS protocol.

**Methods:**

Between 2012–2015, pregnant women opting for the combined test in the North-Netherlands were also invited to participate in a prospective cohort study extending the ultrasound investigation to include a first-trimester ultrasound performed according to a protocol. All anatomical planes included in the protocol were documented by pictures stored for each examination in logbooks. The logbooks of six sonographers were independently assessed by two fetal medicine experts. For each sonographer, logbooks of examination 25-50-75 and 100 plus four additional randomly selected logbooks were scored for correct visualization of 12 organ-system planes. A plane specific score of at least 70% was considered sufficient. The intra-class correlation coefficient (ICC), was used to measure inter-assessor agreement for the cut-off scores. Organ-specific learning curves were defined by single-cumulative sum (CUSUM) analysis.

**Results:**

Sixty-four logbooks were assessed. Mean duration of the scan was 22 ± 6 minutes and mean gestational age was 12+6 weeks. In total 57% of the logbooks graded as sufficient. Most sufficient scores were obtained for the fetal skull (88%) and brain (70%), while the lowest scores were for the face (29%) and spine (38%). Five sonographers showed a learning curve for the skull and the stomach, four for the brain and limbs, three for the bladder and kidneys, two for the diaphragm and abdominal wall and one for the heart and spine and none for the face and neck.

**Conclusion:**

Learning curves for FTAS differ per organ system and per sonographer. Although score-based evaluation can validly assess image quality, more dynamic approaches may better reflect clinical performance.

## Introduction

Prenatal screening for fetal structural abnormalities can be safely performed by ultrasound investigation. A systematic first-trimester anomaly scan (FTAS) at 12–13 weeks of gestation can already detect more than one third of all structural abnormalities and about half of those diagnosed at the second-trimester anomaly scan, with low false-positive rates [1, 2]. The detection rate at the FTAS varies considerably depending on the fetal organ, whether a structured protocol is used, the examination route (transvaginal/transabdominal), the quality of the ultrasound equipment and the sonographer’s experience [3–8]. Evaluation of a sonographer’s experience in the early assessment of fetal anatomy is challenging. Experience and scanning skills are built up over time and criteria to establish when sufficient competence has been reached are lacking. According to the current ISUOG guidelines, sonographers performing FTAS should (1) have completed training in diagnostic ultrasonography and related safety issues; (2) participate in continuing medical education activities; (3) have established appropriate care pathways for suspicious or abnormal findings; and (4) participate in established quality assurance programs [8]. An effective way of visually presenting quality-control and learning curves is by the so-called cumulative summation (CUSUM) analysis, a validated statistical and graphical method displaying shifts in the process mean. The CUSUM analysis is used to assess quality and cumulative performance over a period of time and over a series of recorded measurements [9]. The general idea is that performance can be increased and failures can be diminished by building up experience until an acceptable or predefined level is reached [10]. The CUSUM is widely employed in different fields of medicine [11–13]. In obstetrics it has been recognized as an effective quality-control method to assess arterial Doppler and fetal biometry by ultrasound [14–16]. However, to our knowledge, the evaluation of the learning process of sonographers performing a FTAS using the CUSUM method has not been reported before. Therefore, we set out to evaluate the learning curves of non-novice sonographers performing FTAS as early screening for fetal structural abnormalities. Moreover, we assessed organ-specific scores in order to identify the fetal structures which could potentially impose the biggest challenges for sonographers approaching FTAS. Finally, we evaluated the performance of score-based quality-control for FTAS.

## Methods

### Study design

Between 2012 and 2015, pregnant women opting for the combined test (CT) in the North-Netherlands region were invited to participate in a prospective cohort study offering first-trimester anatomical screening (FTAS), as part of the CT [2]. The systematic assessment of fetal anatomy was based on a protocol including biometric measurements and assessment of anatomical planes. All scans were performed by sonographers (from 5 centers) accredited by the FMF for nuchal translucency (NT) measurement and who had performed at least 100 NT measurements per year. While sonographers were routinely performing NT measurements as part of the combined test, none of them had previously been performing FTAS since this was not included in the national screening program. All sonographers were certified to perform the second-trimester anatomical assessment to scan both transabdominally and transvaginally, as required by the national quality standards for prenatal ultrasound and had completed at least 150 scans per year. Prior to study participation, sonographers received a one-day training aimed at improving their theoretical knowledge on FTAS and their scanning skills. A fetal medicine specialist demonstrated how to obtain the correct scanning planes following the predefined anatomical protocol and discussed detection rates at FTAS. Subsequently the scanning skills of each sonographer were evaluated individually. The following 12 fetal organ systems were investigated: skull, brain, face, neck, diaphragm, heart, abdominal wall, stomach, bladder, kidneys, limbs and spine.

### Research hypothesis

The hypothesis of the study was that a significant difference in image quality and learning curves would be found between the examined fetal organs. We were expecting the lowest scores in image evaluation to be found for the fetal heart. Furthermore, a secondary hypothesis was that overall image quality scores would be mostly graded as sufficient, given the fact that we achieved a high first-trimester detection rate in this study.

### Ultrasound equipment

In the Netherlands sonographers performing NT measurements the second-trimester anatomical assessment are required to work with ultrasound equipment less than five years old and with yearly revision and maintenance. The following quality-standards are set by the National Screening Committee: 17-inch screen, transabdominal and transvaginal transducers, equipped with low (3–5 MHz) and high (7–9 MHz) frequency transabdominal transducers, cine-loop, color Doppler, pulsed wave Doppler, freeze frame and magnification capabilities, electronic calipers, minimum resolution caliper 0.1 mm, digital image-saving and exporting according to the DICOM standards. The examination was always started by transabdominal ultrasound, with the option of switching to transvaginal ultrasound when needed.

### Score-based quality assessment

Throughout the study period, 6 participating sonographers stored all fetal images obtained during each scan and recorded the date, scan’s duration and equipment used. For each sonographer the following was recorded: years worked since FMF accreditation for NT measurement, number of combined tests performed per year and number of second-trimester anomaly scans performed per year (Table 3). When our study was performed, qualification for FTAS was obtained by submitting at least 100 first-trimester scans with nuchal translucency measurement per year, which all of our sonographers did. A minimum of eight FTAS per sonographer were evaluated. These included every 25^th^ scan (25th, 50th, 75^th^, 100^th^etc.), in addition to at least four randomly chosen scans. For sonographers who performed more than 100 scans, each additional 25th scan performed (125^th^, 150^th^, 175^th^ etc.) plus one additional randomly chosen one were analyzed as well.

### Scoring assessment tool

To evaluate the selected logbooks, a scoring assessment tool was developed by a panel of experts, including fetal medicine specialists, researchers and clinical epidemiologists/statisticians. The total score for each organ was obtained by the sum of the single organ-specific items. A total of one, two or three points were allotted to each item. The unequal weighted score was designed to allow for higher scores of the most significant items. In order to test for bias introduced by the unequal weighted scores, all analysis were also performed using a scoring assessment tool assigning 1 point for each correct item (weighted score). After verifying the comparability of the results obtained by the two designs, the unweighted one was chosen. Two qualified fetal medicine specialists (assessor 1 and assessor 2) independently scored each logbook according to a scoring protocol (Table 1). The mean of the two assessors’ scores was used as final score. When multiple images of the same anatomical structure were stored by the sonographers, the image with the highest score was considered for the final score calculation. For each logbook, 12 organ systems were evaluated. An organ-specific score was considered as sufficient when the obtained score was at least 70%.

**Table 1:** Item scoring protocol per organ system.

Fetal organ system	Correct image	Incorrect image
Skull		
• Transversal plane • Cranial bones	1	0
	2	0
Brain		
• Transversal plane • Midline falx • Choroid-plexus-filled ventricles	1	0
	2	0
	2	0
Face—profile		
• Mid-sagittal plane • Nasal bone (measurement) • Nose top, intact lips, mandible • Correct magnification	3	0
	3	0
	1, 1, 1	0
	2	0
Face—Retro-nasal triangle		
• Coronal plane • Two orbits • Retro-nasal triangle	1	0
	2	0
	3	0
Neck		
• Mid-sagittal plane • Correct placing of the calipers • Neutral fetal position • Correct image magnification	3	0
	3	0
	3	0
	2	0
Thorax–Diaphragm		
• Sagittal plane • Diaphragm visible • Heart en stomach visible	1	0
	2	0
	2	0
Thorax—Heart		
• Transversal plane • Four symmetrical chambers • Four chambers–filling with Doppler • V-sign	1	0
	3	0
	3	0
	3	0
Abdominal wall		
• Transversal plane • Insertion umbilical cord	1	0
	2	0
Abdomen—Stomach		
• Transversal plane • Stomach visible	1	0
	2	0
Abdomen—Bladder		
• Transversal plane • Bladder visible • Vessels visible	1	0
	2	0
	2	0
Abdomen–Kidneys		
• Transversal or coronal plane • Both kidneys visible	1	0
	2	0
Limbs*		
• Hands visible • Arms–under arms visible • Arms–upper arms visible	1, 1	0
	1, 1	0
	1, 1	0
• Feet visible • Legs–stand of the foot in sagittal plane • Legs–under legs • Legs–upper legs	1, 1	0
	1, 1	0
	1, 1	0
	1, 1	0
Spine		
• Sagittal plane • Intact overlying skin	2	0
	2	0

*1 point for each limb

### Statistical analysis

Normally distributed variables were described by mean (SD), while skewed distributions were presented by median (range). The unpaired Student’s t-test and Mann-Whitney test were used to test for differences in continuous variables with normal or skewed distributions, respectively. The Chi-Square test was used to test for differences in dichotomous variables. The proportion of correct agreement (95%CI) was used to measure the inter-assessor agreement for all organ-specific scores with a cut-off score of 70%. The intra-class correlation coefficient (ICC, 95%CI) between the assessors was calculated for each of the organ-specific scores. The Landis and Koch criteria were used for the interpretation of the ICC, with K<0: poor agreement, K between 0.0–0.20: slight agreement, K between 0.21–0.40: fair agreement, K between 0.41–0.60: moderate agreement, K between 0.61–0.80: substantial agreement and K between 0.81–1.0: almost perfect agreement) [17]. All analyses (descriptive and comparative statistics) were performed using SPSS version 23 (IBM Corporation, New York, NY, USA). All results were considered statistically significant when p<0.05 (two-sided). Learning curves were designed by the CUSUM chart. The CUSUM score was calculated based on the following equation: CUSUM score = C_t-1_ + (O_t_−E_t_). The CUSUM score is the level of experience up to the current scan, C_t-1_ is the CUSUM score of the previous scan, O_t_ is the observed value of the current scan and E_t_ is the expected value of the current scan. Acceptable failure rate (P_0_), unacceptability failure rate P_1_, Type 1 error rate (α) and type 2 error rate (β) were defined as follows: P_0_ = 10%, P1 = 15%, α = 0.1% and β = 0.05%. For the graphical presentation of the curve, the spacing between the two boundary lines (h) was calculated according to the following formulas: H0 = -b/ (P+Q), H1 = a/(P+Q) where a = ln {(1-β)/ α}, b = ln {(1-α)/ β}, P = ln (p1/p0), Q = ln {(1-p0)/ (1-p1)}, S = Q/(P+Q). A larger t indicates the building-up of experience. Three patterns can be distinguished: 1) the CUSUM scores are stable over time: within the boundary lines but not approaching zero; 2) the CUSUM scores show a learning curve: the line remains within the boundary lines and shows a trend to gradually approaching zero; 3) CUSUM analysis out of control: line falling outside the boundary lines.

### Ethics statement

For the study, a special license was obtained from the ethical committee of the Dutch Ministry of Health, within the Dutch Population Screening Act 11, regulating screening for incurable diseases. The license number is 2014/31. Written informed consent was obtained from all study participants.

## Results

A total of 64 logbooks were assessed. Mean duration of the FTAS was 22.6 ± 6.2 minutes. Table 2 shows maternal and logbook characteristics. Mean maternal age and BMI were 33 ± 4.2 years and 24.8 ± 3.7 Kg/m^2^, respectively and mean gestational age at the time of the scan was 12+6 weeks (range 12+1–13+5) (Table 2). The number of logbooks evaluated for each sonographer ranged between 8 and 15 and the number of logbooks submitted by the sonographer ranged between 100–200. The majority of the scans (60%, n = 38) were performed using high-end ultrasound equipment.

**Table 2:** Study population (n = 64).

Study population	n (%)
**Maternal characteristics**	
Age (years, mean ± SD)	33 ± 4.2
BMI (Kg/m^2,^ mean ± SD)	24.8 ± 3.7
BMI < 25	44 (68.8)
BMI 25–30	17 (26.5)
BMI > 30	3 (4.7)
Gravidity (median, range)	2 (1;3)
Parity (median, range)	1 (0;2)
Gestational age (weeks + days, range)	12+6 (12+1; 13+5)
**Logbooks evaluated for each sonographer**	
Sonographer 1	8 (12.5)
Sonographer 2	9 (14)
Sonographer 3	15 (23)
Sonographer 4	15 (23)
Sonographer 5	8 (12.5)
Sonographer 6	9 (14)
**Total number of logbooks submitted per sonographer**	
Sonographer 1	100
Sonographer 2	125
Sonographer 3	200
Sonographer 4	200
Sonographer 5	100
Sonographer 6	125
**Ultrasound equipment used**	
Mid-range	26 (40.0)
High-end	38 (60.0)

Table 3 shows the characteristics of the participating sonographers. All six sonographers had at least four years of experience with fetal ultrasound and two of them had more than five years. The number of NT measurements performed per year varied between 147–228 while the number of 20-week anomaly scans ranged between 100–1137.

**Table 3:** Characteristics of the sonographers.

Sonographer characteristics at study onset	1	2	3	4	5	6
Years since FMF accreditation for NT measurement	4	4	4	5	5	5
Number of NT measurements performed per year	118	156	148	147	188	228
Number of 20-week anomaly scans performed per year	100*	237	321	1137	103*	356

* This sonographer also performed 20-week anomaly scans in a center for prenatal diagnosis following referral of pregnancies with suspicion of abnormalities. The total number of anomaly scans performed per year was therefore higher than 100.

### Inter-assessor analysis

The results of the inter-observer analysis are presented in Table 4. The agreement level between the two assessors was rated as ‘almost perfect’ for the assessment of the fetal heart, ‘moderate’ for the fetal neck, spine and bladder, and ‘substantial’ for all the remaining organs.

**Table 4:** Agreement analysis per organ system–intraclass correlation coefficient (ICC).

Organ system (Assessor 1)–(Assessor 2)	Proportion correct agreement*	ICC (95%CI)	Agreement level**
Skull	89.0	0.723 (0.545–0.832)	Substantial
Brain	92.0	0.798 (0.666–0.878)	Substantial
Face	75.0	0.722 (0.511–0.838)	Substantial
Neck	73.4	0.560 (0.234–0.762)	Moderate
Diaphragm	76.5	0.736 (0.561–0.841)	Substantial
Heart	87.5	0.907 (0.846–0.944)	Almost perfect
Abdominal wall	80.0	0.794 (0.659–0.876)	Substantial
Stomach	75.6	0.615 (0.363–0.767)	Substantial
Bladder	71.2	0.540 (0.238–0.723)	Moderate
Kidneys	75.0	0.681 (0.469–0.808)	Substantial
Limbs	77.2	0.735 (0.560–0.840)	Substantial
Spine	75.0	0.548 (0.263–0.724)	Moderate

* Based on cut-off for correct agreement of 70%. All results show p<0.05

**Based on the Landis and Koch criteria for agreement for ICC [17]

<0 Poor agreement

0.0–0.20 slight agreement

0.21–0.40 fair agreement

0.41–0.60 moderate agreement

0.61–0.80 substantial agreement

0.81–1.0 almost perfect agreement

### Organ-specific learning curves

**Fig 1:**
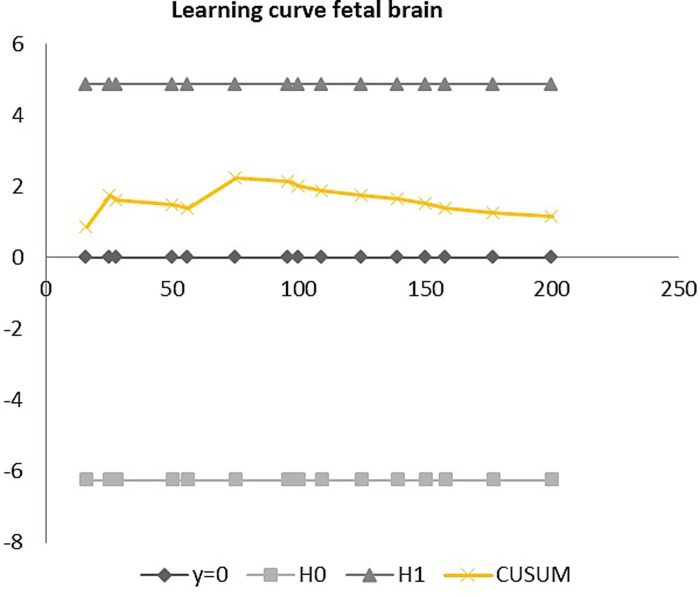
CUSUM analysis showing a learning curve for the fetal brain. Where y = 0 represents the 13-week anomaly scans performed by the sonographers throughout the study period. H0 and H1 represent the lower and upper limits of the CUSUM graph, which should not be crossed by the CUSUM plot (in yellow) for the process to not go out of control. CUSUM represents the learning curve obtained by CUSUM analysis, where a decreasing slope indicates a positive learning process and thus an improvement in performance, as in this case.

**Fig 2:**
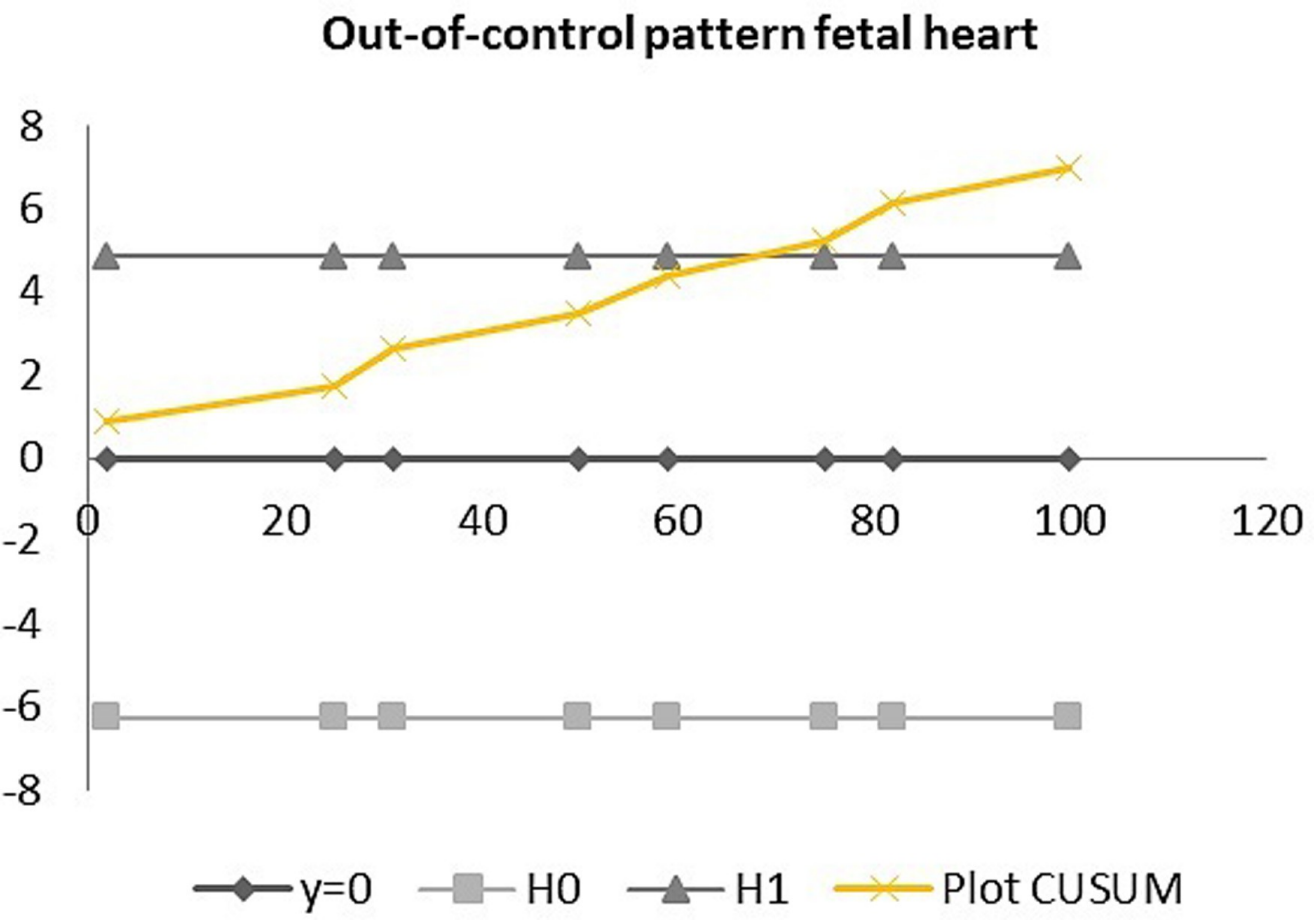
CUSUM analysis showing an out-of-control pattern for the fetal heart. Where y = 0 represents the 13-week anomaly scans performed by the sonographers throughout the study period. H0 and H1 represent, respectively, the lower and upper limits of the CUSUM graph, which should not be crossed by the CUSUM plot (in yellow) for the process to not go out of control. CUSUM represents the learning curve obtained by CUSUM analysis. In this case the CUSUM line crosses the upper limit, therefore showing an out of control pattern and thus no clear change in performance.

Table 5 shows the proportion of images with a sufficient score (> = 70%) obtained by each sonographer for each organ system. Sonographer 5 achieved the highest proportion of sufficiently graded logbooks (65%), while the lowest proportion was obtained by sonographer 3 (47%). When looking at the 6 sonographers altogether, 57% of the collected logbooks were graded as sufficient. The highest proportion of sufficient scores was obtained for the fetal skull (88%), brain (70%) limbs (69.5%) and kidneys (69%), while the lowest scores were for the fetal face (29%), spine (38%) and neck (39%). Table 6 summarizes the results of the organ specific CUSUM analysis. Five of six sonographers showed a learning curve for the assessment of the fetal skull and stomach. Four sonographers showed a learning curve for the examination of the brain and limbs. Three sonographers showed a learning curve for the examination of the fetal bladder and kidneys. Two sonographers showed a learning curve for the fetal diaphragm and abdominal wall. One sonographer showed a learning curve for the assessment of the fetal heart and spine. For the fetal face and neck, we did not observe any learning curves amongst the six sonographers. An out-of-control pattern was observed in 4 of the 6 sonographers for the face, diaphragm and spine and in 3 for the heart and bladder. Two graphic examples of CUSUM results can be seen in Figs 1 and 2, representing a learning curve and an out-of-control pattern respectively.

**Table 5:** Proportion of logbooks with acceptable scores (> = 70%) per organ system and per sonographer.

Sonographer	Skull	Brain	Face	Neck	Diaphragm	Heart	Abdomen wall	Stomach	Bladder	Kidneys	Limbs	Spine	Total
**1**	100	87.5	50	50	25	0	50	87.5	75	75	75	37.5	**60**
**2**	50	50	37.5	37.5	62.5	37.5	37.5	100	50	75	75	25	**53**
**3**	94	62.5	18.7	18.7	31.2	12.5	75	56	18.7	68.7	68.7	37.5	**47**
**4**	93	73	33	67	20	67	46.7	80	87	60	60	47	**61**
**5**	100	70	0	28	71	57	85	100	71	57	71	71	**65**
**6**	89	78	33	33	33	100	67	88	22	78	67	11	**58**
**Total**	**88**	**70**	**29**	**39**	**40.5**	**46**	**60**	**85**	**54**	**69**	**69.5**	**38**	**57**

**Table 6:** Organ-specific CUSUM analysis.

Sonographer	Skull	Brain	Face	Neck	Diaphragm	Heart	Abdominal wall	Stomach	Bladder	Kidneys	Limbs	Spine
**1**	**curve**	stable	stable	stable	X	X	stable	**curve**	**curve**	**curve**	**curve**	X
**2**	stable*	stable	stable	stable	**curve**	X	X	**curve**	X	stable	**curve**	X
**3**	**curve**	**curve**	X	X	X	X	**curve**	X	X	X	stable	X
**4**	curve**	**curve**	X	stable	X	stable	X	**curve**	**curve**	stable	**curve**	X
**5**	**curve**	**curve**	X	X	**curve**	stable	**curve**	**curve**	**curve**	**curve**	**curve**	**curve**
**6**	**curve**	**curve**	X	stable	X	**curve**	stable	**curve**	X	**curve**	stable	stable

*Stable: CUSUM analysis showing stable curve over time.

**Curve: CUSUM analysis showing learning curve.

X: CUSUM analysis showing out-of-control pattern.

### Item scores

Table 7 shows the percentages of images with correctly shown anatomical landmarks, scanning planes and image magnification per organ system. The detailed item scores can be found in Table 7. The skull and brain had the highest scores for correct anatomical landmarks (skull: 92.1%, brain: 80.2%) as well as for correct scanning planes (skull: 98.4%, brain: 96.8%), while the score for correct image magnification was the highest for the neck (79.4%) and limbs (79.0%). The lowest scores for correct anatomical landmarks were for the heart (30.3%) and the profile (38.9%). The lowest scores for correct scanning planes were for the diaphragm (34.9%), the neck (38.1%) and the profile (39.7%).

**Table 7:** Item scores for the evaluation of the ultrasound images per organ system.

Fetal organ	Correct anatomical landmarks (%)	Correct scanning planes (%)	Correct image magnification*(%)
Skull	92.1	98.4	71.4
Brain	80.2	96.8	69.8
Face—Profile	38.9	39.7	84.1
Face—Retronasal triangle	52.5	55.6	55.6
Neck	55.6	38.1	79.4
Diaphragm	45.2	34.9	39.7
Heart	30.3	85.7	65.1
Abdominal wall	68.3	61.9	46.0
Stomach	81.0	97.3	60.0
Bladder	56.3	60.3	38.1
Kidneys	57.1	60.8	54.0
Limbs	49.5	61.9	79.0
Spine	44.4	55.6	61.8
Total	57.8	65.1	61.9

*Correct magnification was not included as scoring criterion in the anatomical assessment protocol

### Subgroup analyses

We did not find any correlations between organ-specific scores and ultrasound duration (p>0.05). In our cohort most women (68.8%, n = 44) had a BMI<25 Kg/m^2^, 26.5% (n = 17) had a BMI between 25–30 Kg/m^2^ and 4.7% (n = 3) had a BMI >30 Kg/m^2^. We did not find any significant correlations between the BMI group (<25, 25–30 and >30 Kg/m^2^) and each obtained organ-specific score (p>0.05). Ultrasound duration (in minutes) was also not correlated to maternal BMI (p = 0.6). All ultrasounds were performed transabdominally. The use of a high-end ultrasound machine was correlated to higher scores for the fetal heart (p<0.002) but not for all other fetal organs.

## Discussion

This study reports on the quality of ultrasound images obtained by sonographers performing a systematic first-trimester anomaly scan. All sonographers were FMF-certified for NT measurement and experienced with the second-trimester anomaly scan. The aim of the study was to evaluate the quality of the ultrasound images by item grading and to establish whether a learning curve could be observed for non-novice sonographers undertaking this new ultrasound screening. Logbooks were scored as of sufficient quality (≥70%) in 57% of the analyzed cases. The proportion of images with sufficient scores varied considerably between fetal organs and was the highest for the skull (88%) and brain (70%) and the lowest for the spine (29%). A learning curve by CUSUM analysis was identified most frequently for the correct visualization of the fetal skull, stomach, brain and limbs. Whereas the organs more often presenting an ‘out-of-control’ pattern were the diaphragm, spine, heart and bladder. These same organs also showed the lowest proportion of images with sufficient scores. While for the fetal heart this could be due to the technical difficulty of early fetal cardiac examination, the finding was more surprising for the spine. The suboptimal image quality could also explain the moderate agreement between the two assessors for the evaluation of the fetal spine, an image more prone to subjective judgment. Hence, only 56% of the images displayed the fetal spine in a correct sagittal plane and only 44% clearly showed the overlying skin. It was surprising to note that only 39% of the logbooks documenting the fetal neck were scored as sufficient, considering that all sonographers were FMF-certified and experienced in NT measurement. Although the technical difficulty of accurate NT measurement in a clinical setting has been previously reported, the fact that only 38% of the documented images showed a correct mid-sagittal plane remains of concern [18, 19]. The use of a prospective ongoing quality assessment with personalized feedback for the operator has been effective in improving performance in both NT measurement and second-trimester anomaly scans [20, 21]. However, these approaches are time-consuming and labor-intensive and might be challenging to implement, especially when the image evaluation is not restricted to a single plane [19, 22]. The CUSUM analysis is a recognized, intuitive and sensitive method to successfully monitor and audit the quality of, for instance, NT measurement and document a learning curve [23]. However, a limitation of this method is that once the trend line shows an out-of-control pattern, it fails to quickly return between the upper and lower limits. The fact that a significant proportion of out-of-control cases was found in the organ-systems with the poorest scores (i.e., spine, heart, face, diaphragm) could indicate that the CUSUM-methodology might have failed in demonstrating the learning process of images with lower quality. Indeed, the CUSUM-design relies on the chosen acceptability cut-off, which was 70% in this study. Therefore, all images with a score below the chosen cut-off are identified as ‘unacceptable’ and seen as lack of improvement in performance, without further describing the degree of ‘unacceptability’ of the given score. Another possible explanation for the high proportion of out-of-control patterns could be identified in the number of chosen measured time points (8–16), which may have been too little to correctly identify improvements in sonographers’ performance. Moreover, while the unequal number of examined logbooks for each sonographer was chosen to allow for longer observation of the learning process in sonographers who performed a higher number of FTAS during the study period, this methodology might have introduced some sampling bias.

Factors such as sonographers’ experience, scanning conditions and ultrasound equipment are also known to influence performance [24]. We did not find any association between high maternal BMI (≥30 Kg/m^2^) and poor image quality on transabdominal ultrasound. However, this could be due to the low number of women (n = 3) with a BMI≥30 Kg/m^2^. We were able to confirm the previously described effect of ultrasound equipment characteristics on fetal cardiac assessment [25]. Other factors potentially affecting image quality are gestational age, time constraint and sonographers experience [19, 26]. A limitation of the study is that logbook evaluation should have ideally occurred prospectively. This would have allowed us to monitor the effects of a given feedback on the performance of the sonographers.

In spite of the low proportion of logbooks with sufficient scores, the detection rate of structural abnormalities in this study was extremely high, reaching 100% for the anomalies amenable to first trimester diagnosis [2]. This apparent paradox indicates the mismatch between image quality and true detection rates. Score-based evaluation appears to be a valid tool for the assessment of image quality, as suggested by the high level of agreement between the two assessors, who were hence able to discern images with adequate quality from the poor ones. However, it might not accurately reflect true detection rates in clinical practice. Indeed, at this early gestation the fetus is very active and documenting anatomical planes on static images may be far more challenging and time-consuming than confidently assessing their normality during the scanning process. For instance, it is by far easier to exclude a large abdominal wall defect, a megacystis or a large myelomeningocele during the scanning process, than to store an optimal image of the same anatomical regions when no anomalies are seen. At present, the main goal of the FTAS is to detect severe and lethal abnormalities. A more advanced examination of other anatomical regions such as the fetal profile and the heart, or the use of the transvaginal approach may in the future increase detection of less severe abnormalities, but for the time being, adherence to a protocol aimed at excluding severe anomalies will serve the main purpose of the screening, i.e., offering parents the option of early diagnosis of severe, mostly lethal, abnormalities. In this context quality-control by static image evaluation may therefore fall short in truly reflecting the performance of the FTAS. The use of artificial intelligence, although still experimental and of simulation-based learning may be a far more effective method to monitor the performance of sonographers novice to first trimester anatomical screening and improve their scanning skills in a cost-effective way [27–30].

## Conclusion

Learning curve of sonographers performing FTAS show different patterns based on the operator and the fetal organ assessed. Although the CUSUM method was able to show learning curves for some organ systems, future studies with larger cohorts, longer longitudinal observation and a prospective design are needed to further evaluate the learning process of sonographers performing FTAS. Finally, although score-based evaluation seems to be a valid tool for the assessment of static image quality, more dynamic approaches may be more appropriate to reflect true clinical performance and detection rate.
